# Children’s Preference for Causal Information in Storybooks

**DOI:** 10.3389/fpsyg.2020.00666

**Published:** 2020-04-15

**Authors:** Margaret Shavlik, Jessie Raye Bauer, Amy E. Booth

**Affiliations:** ^1^Department of Psychology and Human Development, Vanderbilt University, Nashville, TN, United States; ^2^Department of Psychology, University of Texas at Austin, Austin, TX, United States

**Keywords:** early literacy, shared reading, book preference, causal information, causal stance

## Abstract

Fostering early literacy depends in part on engaging and inspiring children’s early interest in reading. Enriching the causal content of children’s books may be one way to do so, as causal information has been empirically shown to capture children’s attention. To more directly test whether children’s book preferences might be driven by causal content, we created pairs of expository books closely matched for content and complexity, but with differing amounts of causal information embedded therein. Three and 4 years old participants (*n* = 48) were read both books and their interests and preferences were evaluated. When asked to choose, children preferred the highly causal over the minimally causal books. Results are discussed in terms of broader implications for creating books that optimally engage young children, as well as guiding book selections parents and educators make in their endeavors to promote interest in reading and early literacy.

## Introduction

From a young age, children have a strong interest in discovering the causal structure of the world around them. For this reason, Piaget (1952) described children as “little scientists,” tirelessly exploring and seeking explanations. More contemporary theorists echo this characterization, further emphasizing the intrinsically rewarding experience of causal discovery (Gopnik, 1998), and identifying potential physiological underpinnings thereof (Biederman and Vessel, 2006).

Children’s interest in causality (e.g., functional revelations or explanations regarding the why and how of the world) has already been demonstrated across numerous experimental settings. For instance, children will spontaneously and persistently ask questions about novel objects until causally-relevant information is revealed (Greif et al., 2006; Alvarez and Booth, 2016). Children also explore novel toys longer if their causal structure is ambiguous or contradictory than if it is expected (Bonawitz et al., 2011; Cook et al., 2011; Marcis and Sobel, 2017). Still other work reveals that young children will persist longer at a tedious motor task when rewarded with causally rich rather than minimally causal descriptions of novel objects (Alvarez and Booth, 2014), and tend to favor informants who have a history of providing causal information (Sobel and Corriveau, 2010). Evidence suggests that this interest in causal information is not a mere byproduct of children’s interest in discovery generally-speaking, as young children have been shown to prefer causal revelations above and beyond other types of non-obvious information about the world (Alvarez and Booth, 2015).

Although this body of research compellingly demonstrates children’s early attunement to causal information, it is limited by the fact that each contributing study was conducted in a highly contrived context. For example, in Alvarez and Booth (2015), preschoolers were presented with two identical puppets that offered either causally rich or minimally causal descriptions of novel items. When children were asked on each trial to indicate which puppet they would like to learn from, they most often selected the former. But this is an odd situation. Children are rarely (if ever) confronted with the need to choose between distinct informants in this manner, let alone *puppet* informants. Perhaps this unnatural context artificially heightened attention and sensitivity to the descriptions offered. Thus, although this work is theoretically informative, it remains unclear whether children’s preference for causal information is sufficiently robust to guide behavior in more realistic contexts.

Confirming the generalizability of children’s causal stance is important because attunement to causal information has developmental consequences (Mantzicopoulos et al., 2008; Stahl and Feigenson, 2015; Bauer et al., 2016). Perhaps most intuitively, interest in the causal properties of objects, and animals might be fundamental to children’s engagement in scientific inquiry in both naturalistic and academic learning environments (Conezio and French, 2002; Legare, 2014). However, its relevance might be considerably broader than this. Children’s causal stance might, for example, also be a potent motivating factor in the context of shared-book reading, potentially driving children’s selection of books, and boosting engagement in the reading process.

Shared book reading, especially Dialogic Reading (Whitehurst et al., 1994), is known to facilitate early print awareness, vocabulary, and ultimately reading skill, in young children (Bus et al., 1995; Ezell and Justice, 2005; Sénéchal, 2010; Farrant and Zubrick, 2012). But to maximize the impact of shared book reading on early literacy, it is crucial to foster children’s affinity for this activity (Holdaway, 1979; Whitehurst et al., 1988; Lyytinen et al., 1998). The relationship between the desire to read and literacy development is remarkably consistent (Krashen, 1993), with children’s engagement in reading sometimes even correlating to later literacy more robustly than other theoretically strong predictors like reading frequency or type of parent reading (Crain-Thoreson and Dale, 1992). Importantly, Pezoa et al. (2019) further demonstrate that child interest in reading is more predictive of parents’ book reading practices than the other way around. Together, this work reinforces the importance of optimizing children’s enthusiasm for reading.

Multiple factors might influence young children’s interest in reading particular books. In terms of illustrative style, preschoolers seem to prefer picture books with familiar images that are representational (vs. abstract) and colorful (Danko-McGhee and Slutsky, 2011). In terms of story genre, two recent studies also suggest that young children have a broad preference for expository (i.e., informational) over narrative books (Kotaman and Tekin, 2017; Robertson and Reese, 2017). One possibility is that it is the causal content embedded in expository texts that particularly captures children’s interest and drives these preferences. Both narrative and expository texts can of course reveal the causal structure of the world, but only the latter are typically designed to do so in an explicit manner. Unfortunately, because the narrative and expository texts offered to children in these previous studies potentially varied on a number of dimensions related to their distinct genres, they cannot speak directly to the question of whether differences in causal content were responsible for the observed preferences.

In order to eliminate these extraneous differences, and to thereby address the question of causality-focused interests with greater precision, we focus on preferences between books drawn from a single genre. Although we could have chosen any genre in which to implement our manipulation of book content, our goals were most readily achieved by focusing on expository texts which frequently, but not universally, incorporate the type of causal explanatory information of interest in this study. Specifically, we take advantage of natural variability in the degree to which causal information is written into expository books by comparing children’s preferences for an expository book rich with causal explanations regarding why animals behave the way they do, against an expository book that included only perceptual descriptions of animals.

Based on the evidence reviewed above, we hypothesized that the causally rich book content would be particularly compelling to children and thus would influence their preferences. Importantly, this is not a foregone conclusion. It is entirely possible that children are not sensitive to the causality of information presented in the otherwise engaging context of shared book reading. Indeed, the high level of stimulation inherent in reading novel books with an attentive adult could overwhelm children’s sensitivity to qualitative differences in book content. A controlled examination is therefore critical for disambiguating these possibilities.

## Materials and Methods

### Participants

Our sample included 48 children (21 female) from the Austin, Texas area. Participating children were 3–4 years old (*M* = 4;0, *SD* = 0;3, range = 3;3–4;7). Children were recruited through an existing database of families interested in participating in research. Children did not have any diagnosed developmental disorders or hearing impairments, and spoke English more than 50% of the time at home (based on parent report). An additional fourteen children were excluded from analyses due to attrition after the first visit (*n* = 6), noncompliance with study tasks (*n* = 3), prior exposure to the books used as stimuli (*n* = 2), and experimenter error (*n* = 3).

Based on parent report, 10.4% of participating children were African American, 72.9% were White, 4.2% were Asian, and 12.5% identified as multiple races or “other.” In addition, 31% of these children were also identified by their parents as being Hispanic or Latino. While our sample closely reflected the racial and ethnic composition of the Austin metropolitan area (and was not dissimilar to the U.S. Population overall), the majority of our families were from middle-to high socioeconomic backgrounds. With respect to maternal education, 8.3% held a high school degree, 8.3% completed some college or additional training beyond high school, 48.0% had a 4-year bachelor’s degree, and 35.4% held a master’s degree or higher.

### Materials

Because animals are among the most popular subjects of children’s books (Marriott, 2002) and clearly garner children’s interest from a young age (DeLoache et al., 2011), we selected two expository children’s books about animals. Although both were authored and illustrated by Steve Jenkins, and targeted the same-aged audience, they differed in the degree to which they emphasized causal information. “Biggest, Strongest, Fastest” (Jenkins, 1997) contained minimally causal information, while “What Do You Do When Something Wants to Eat You?” (Jenkins, 2015) contained a wealth of causally rich information. More specifically, the former explained on each page how a particular behavior or body part was relevant to the pictured animal’s survival, whereas the latter provided factual, but non-explanatory, descriptions. Although causal properties could certainly be inferred from the descriptions and pictures provided in the minimally causal “Biggest, Strongest, Fastest,” they were never explicitly stated.

We chose to use these existing picture-books as stimuli in order to maximize the ecological validity of our findings. However, this necessarily meant sacrificing the precise experimental control over book content that could have been achieved by writing our own books from scratch. Although the two Steve Jenkins books we selected were well-matched in terms of illustrative and textual style, as well as their general topical content (animal characteristics), they differed in other ways. For example, the specific animals pictured were different in the two books, and “What Do You Do When Something Wants to Eat You?” contained more words per page (25.5 vs. 9.4 in “Biggest, Strongest, Fastest” averaged just 9.4). Although there is no clear theoretical reason to believe that these differences would sway children’s preferences, we felt it was important to address them. In order to do so, we created a second version of each book by editing each page to contain the inverse amount of causal information, while maintaining the original illustration and text length (see Table 1). In the edited pair, “Biggest, Strongest, Fastest” therefore became the causally rich book, while “What Do You do When Something Wants to Eat You?” became the minimally causal book. The four book versions are summarized in Table 2. Half of the participating children were read the original pair of books as written by Steve Jenkins, and the other half were read the edited counterparts.

**TABLE 1:** Example sentences from the original and rewritten texts.

	Original	Rewrite
“What Do You Do When Something Wants to Eat You?”	The hover fly is a harmless insect without a sting. But it can protect itself from predators by mimicking the appearance of a wasp.	The hover fly looks like a bumblebee with its black and yellow body. But if you look closely, you can see it has no stinger.
	If a puffer fish is in danger… it takes in water and swells up like a prickly balloon, making itself almost impossible to swallow.	If you see a pufferfish in the ocean… its puffy body will be filled up with water and covered with lots of prickly little spikes.
“Biggest, Strongest, Fastest”	The sun jellyfish is the world’s longest animal.	The sun jellyfish catches food with its long tentacles.
	The flea is very small, but it is the world’s best jumper.	The flea has bendy legs to jump way up high.

**TABLE 2:** Book pairs.

	“What Do You Do When Something Wants to Eat You?”	“Biggest, Strongest, Fastest”
Pair 1 (original versions)	Causally rich	Minimally causal
Pair 2 (rewritten versions)	Minimally causal	Causally rich

*Half of the participants were read Pair 1 at both sessions while the other half were read Pair 2 at both sessions.*

### General Procedure

This study involved two sessions, spaced ∼2 weeks apart. At each session, a female experimenter read a pair of books to the child. The very same two books were read both times, but their order of presentation was counterbalanced across sessions. After reading each book, the child rated how much they enjoyed reading the book and answered five comprehension questions. Lastly, the child chose which of the two books he or she preferred. Both sessions took place in a quiet room with minimal distractions at our research lab on the University’s campus. Parents consented for both themselves and their child by signing a single consent form in person. All sessions were audio-visually recorded for offline coding. Upon completion of each visit, the family was compensated, and each child was given a book to take home.

### Procedure: Visit 1

#### Book Reading

Each child was read one pair of books: one version of each title (“Biggest, Strongest, Fastest” and “What Do You Do When Something Wants to Eat You?”). One book was causally rich and the other was minimally causal (see Table 1). The order of presentation was counterbalanced across participants. At the second visit, the child was read the same pair of books, but in the opposite order. A single experimenter read all books (across sessions) to any particular child. Because interactive (e.g., dialogic) book reading is known to facilitate child engagement, and to support story comprehension, it was important to standardize the style with which experimenters read with the children. In order to minimize distraction from the book content (in which our manipulation was embedded) experimenters avoided extratextual talk entirely, and only read the words on the page. If the child asked questions, the experimenter redirected them to the book with neutral statements such as, “let’s see what the book says next!” She also used simple gestures that were kept consistent in both the causally rich and minimally causal versions of each book (e.g., circled an animal with her finger as she described it).

#### Book Rating

After reading each book, the experimenter asked the child to rate how much they liked the book according to a 5-point Likert-like “Smiley-Face Scale” (adapted from the Wong-Baker FACES Scale; Wong and Baker, 1988). We included this measure in hopes that it would provide a convergent measure of children’s preferences. However, despite evidence suggesting that young children can respond appropriately to this type of scale (e.g., Macklin and Machleit, 1990), 75% of our participants failed to pass the training portion of this procedure (i.e., they failed to differentially rate a wide range of items and/or rated clearly undesirable items at the highest end of the scale) during one or both testing sessions. As a result, we unfortunately were unable to meaningfully utilize this data.

#### Comprehension Questions

Five comprehension questions were administered after reading each book to ensure the child was paying attention and understood the content (see Supplementary Appendix for an example). Each question was accompanied by two illustrations excerpted from the books. Children selected their answer to each question by pointing to, or verbalizing, their choice. It is important to note that while the questions were designed to be relatively easy for a child who read the book, the answers were not obvious from simply looking at the two pictures. The experimenter thanked the child for their responses but did not provide corrective feedback.

#### Measuring Explicit Book Preference

Once both books were read, the child was given a short break. Afterwards, the experimenter placed both books in front of the child, read both titles, and asked them which book they liked more. If the child was hesitant at first, the experimenter rephrased the question by asking, “Which one was your favorite?” To ensure that the experimenter did not bias responding, she presented the books in the order in which they were read (which was counterbalanced across sessions) and looked only at the child (i.e., not at either of the books) when asking for their preference.

### Procedure: Visit 2

Two to four weeks after their first visit, parent-child dyads returned to the laboratory for a second session. The procedure mirrored the first session, with two exceptions. First, although children read the same two books as before, they were presented in reverse order, such that they heard the causally rich book first in one session and the minimally causal book first in the other. Second, the story comprehension questions were rephrased so that the distractor pictures from questions at the first session were now the correct answers. This was done in order to control for any potential intrinsic appeal of a particular pictured response option.

### Coding

Participant data were coded and managed using Research Electronic Data Capture (REDCap; Harris et al., 2009). REDCap is a secure, web-based application designed to support data capture for research studies. Book ratings, story comprehension, and book preference were coded offline. A second reliable research assistant also coded 20% of the videos to ensure reliability. No discrepancies were detected.

Videos were also coded offline for children’s overall engagement during the book reading activity. Pilot data demonstrated that this was most reliably measured using a flexible 3-point scale (rather than on the basis of specific indicators that manifested inconsistently across children). The scale differentiated between low, moderate, and high levels of engagement. A primary coder rated 100% of participant videos and a blind secondary coder scored 20% of the videos. Overall, there was excellent agreement between coders (Cohen’s kappa = 0.96).

## Results

Children performed similarly on comprehension questions for causally rich (*M* = 4.04, *SD* = 0.97) and minimally causal (*M* = 4.05, *SD* = 0.90) books, *t*(47) = −0.074, *p* = 0.94, confirming that they were well matched in terms of the accessibility of their content and thereby discounting the possibility that any observed book preferences were due to better understanding of one book over the other. A uniformly high level of enthusiasm during book reading sessions was evident in the 3-point global ratings of engagement (*M*_*rich*_ = 2.24; *SD* = 0.66, *M*_*minimal*_ = 2.36, *SD* = 0.62), *t*(47) = −1.60, *p* = 0.12.

We compared children’s explicit book preferences (for title and for causal content) across both sessions using two chi-square goodness-of-fit tests (see Table 3). In general, there was an effect of book title (children preferred “What Do You Do When Something Wants to Eat You?” over “Biggest, Strongest, Fastest),” χ^2^(2, *N* = 48) = 13.46, *p* = 0.001. This confirms the importance of counterbalancing causal content within titles in this experimental design. By doing so, we are able to analyze with clarity whether children prefer the causally rich content, despite other factors affecting their interest, like the appeal of the title, or the specific animals pictured throughout.

**TABLE 3:** Explicit book choices combined across sessions.

	Frequency of book choice patterns		
	Minimally causal (at both visits)	One of each	Causally rich (at both visits)
Observed	14	13	21
Expected (chance)	12	24	12

*Children’s observed preference for causal books was significantly larger than what would be expected if they were merely choosing at chance levels. Note that the “one of each” category collapses the two ways that children could have chosen a combination (causally rich at session 1 and minimally causal at session 2, or vice versa).*

Note that there were four possible patterns according to which children might have selected books across the two sessions (causally-rich/causally-rich, causally-rich/minimally-causal, minimally-causal/causally-rich, minimally-causal/minimally-causal). However, because two of these (causally-rich/minimally-causal, minimally-causal/causally-rich) were equivocal with respect to children’s preferences, there were actually only three meaningfully distinct response patterns. Based on chance, we would expect 25% of children to choose the causally-rich option at both sessions, 25% to choose the minimally causal option at both sessions, and 50% to choose each book once across the two sessions. As described in Table 3, our observed findings differed significantly from this pattern, χ^2^(2, *N* = 48) = 12.13, *p* = 0.002, and the size of the effect was relatively large (ω = 0.50). Indeed, 43.75% of children chose the causally rich book at both sessions, 27.08% chose the causally rich book at just one session (either Visit 1 or 2), and 29.17% chose the minimally causal book at both sessions. A follow-up binomial test confirmed that the number of children who selected the causally rich book during both visits was greater than the chance value of 25%, *p* = 0.004.

## Discussion

This investigation focused on the intersection between existing research on young children’s attunement to the causal structure of the world and on children’s book preferences. Specifically, we asked whether children’s book preferences might be driven, at least in part, by their interest in causality. The core finding revealed by this investigation was that young children do indeed prefer storybooks containing causally rich information to those containing minimally causal information. Importantly, this result emerged even though comparison books were matched carefully in terms of text complexity, length, illustrative quality, and comprehensibility.

This finding is consistent with prior research and theory detailing young children’s “causal stance,” or early emerging motivation to acquire causally-relevant knowledge (e.g., Gopnik, 2000; Alvarez and Booth, 2014). In particular, the current work parallels Alvarez and Booth (2015), in which preschoolers explicitly chose to learn about the causal powers of objects and animals over other types of information. One key strength of this study is that it extends the generalizability of these findings to a somewhat more realistic setting: shared book-reading. Given all of the distractions that might capture a young child’s attention in this context (e.g., the novel laboratory setting, the unfamiliar experimenter, a new book packed with bold illustrations), it is remarkable that children detected the key qualitative difference in causal content, and used it to guide their book selections.

The current findings also relate to existing research suggesting that children generally prefer expository texts over narrative texts (Kotaman and Tekin, 2017; Robertson and Reese, 2017). To the extent that the former typically contain more information about the causal structure of the world than the latter, the preference for causally rich books observed here is well aligned with this finding. However, it should be noted that, on average, narrative storybooks might actually be *richer* than expository texts in other types of causal information, specifically with respect to psychological themes or story structure. One limitation of the current is that it focused exclusively on expository texts and behavioral/functional causality, thereby precluding any strong conclusions about the relative pull of different types of information embedded in different types of books. Future work will therefore be necessary to systematically test the basis for children’s book preferences across different genres and types of causality.

Other limitations of the current study spotlight additional important directions for future work. For example, it will be important to explore whether children’s preference for causally rich content is maintained when books are not explicitly pit against each other. We attempted to take a step towards addressing this question with our smiley-face rating task, but given the universally positive responses we observed, this was clearly not a sufficiently sensitive measure for this sample of children. Recall that we also coded children’s engagement during the book reading session and found consistently high levels regardless of book type. This might be taken as evidence that children would not express preferences if not forced to choose among types of books. However, it might also be that children were more broadly excited by the playful lab setting, new books, and the attentive adult reader. To overcome these limitations, future work might focus on recording parents reading books to their child in their home, thus minimizing irrelevant stimulation and further increasing ecological validity. Following Robertson and Reese’s work (2017), families could be given several books that systematically vary on key dimensions to assess children’s preferences.

Another limitation of note, derives from our decision to utilize existing children’s books as stimuli. Although this approach contributes positively to the ecological validity of the work, it constrained our ability to control for all potentially important dimensions of the causally-rich and minimally causal comparison books simultaneously. One confound of particular interest might be the degree to which the texts embody dynamic action as opposed to static description. By its very nature, causal information necessarily refers to some form of agentive action or transformation. Non-causal descriptions are not constrained in this way, and can be completely devoid of action. And indeed, as a rough index, there were somewhat more verbs in the causally rich texts utilized in this study (average of 3 per description) than in the minimally causal versions (average 2.22 per description). Therefore, it might be possible that children’s preference for causally rich texts is driven by a more basic preference for dynamic action. While we are unable to explicitly test this possibility in the current study, the trial-by-trial responses of preschoolers tested by Alvarez and Booth (2015) were not affected by the degree of dynamic information embedded in item descriptions provided by puppet informants. Although this related data suggests that dynamic information is unlikely to wholly account for the book preferences observed in the current work, a more careful examination of the respective influence of these dimensions could be attained by better isolating them in books written from scratch.

It will also be interesting to specify, in future work, whether children’s preferences for causally rich books translates into superior learning. Causal explanations have already been shown to support the acquisition of knowledge in other settings (e.g., Gopnik and Sobel, 2000; Booth, 2015; Bauer et al., 2016). For instance, in one study, preschoolers recalled more novel labels for unfamiliar objects or animals after a delay when they were accompanied by causal descriptions than when they were accompanied by non-causal descriptions (Booth, 2009). Although the current study tested children’s knowledge of book content, these questions were intended only as a basic comprehension check. More difficult questions (perhaps again focusing on novel vocabulary) would be necessary to gain sufficient sensitivity to variations in learning across levels of causal richness in books.

Although much remains to be done in this area of inquiry, the current work lays a solid foundation for exploring the potential relevance of children’s causal stance to real-world learning contexts. Specifically, it demonstrates that children’s interest in causal information extends to personal book preferences. This insight could be useful to parents, educators, and authors working to facilitate early literacy. By choosing optimally engaging books, the documented benefits of shared book reading could further scaffold young children’s oral language and literacy skills (Bus et al., 1995; Hargrave and Sénéchal, 2000; Sénéchal, 2010; Farrant and Zubrick, 2012).

## Data Availability Statement

The raw data supporting the conclusions of this article will be made available by the authors, without undue reservation, to any qualified researcher.

## Ethics Statement

The studies involving human participants were reviewed and approved by University of Texas IRB. Written informed consent to participate in this study was provided by the participants’ legal guardian/next of kin, and verbal assent was provided by the participating child at the start of each session.

## Author Contributions

MS, JB, and AB contributed to the design and implementation of the research, to the analysis of the results, and to the writing of the manuscript.

## Conflict of Interest

The authors declare that the research was conducted in the absence of any commercial or financial relationships that could be construed as a potential conflict of interest.
