# Supplementary material for: Children’s Preference for Causal Information in Storybooks
Source: Front Psychol. 2020 Apr 15;11:666. doi: 10.3389/fpsyg.2020.00666 (PMC7174774; doi:10.3389/fpsyg.2020.00666)
Supplement: Supplementary file 1 [file Table_1.DOCX]

Appendix

*Example comprehension questions*

|  |  | Causally Rich  Version | Minimally Causal Version | Picture Choices  (L, R; *correct*) |
| --- | --- | --- | --- | --- |
| Visit 1 | “What Do You Do When…?” | Which one swells up like a balloon when it is in danger? | Which one’s body is filled with water and covered with lots of spikes? | hog-nosed snake, *pufferfish* |
|  | “Biggest, Strongest, Fastest” | Which one hides in its shell for protection? | Which one is one of the slowest animals? | *snail*, jellyfish |
| Visit 2 | “What Do You Do When…?” | Which one plays dead when it feels threatened? | Which one did the book say has black and brownish patches on its back? | *hog-nosed snake*, pufferfish |
|  | “Biggest, Strongest, Fastest” | Which one catches food with its tentacles? | Which one is one of the longest animals? | snail, *jellyfish* |
